# Plant recording across two centuries reveals dramatic changes in species diversity of a Mediterranean archipelago

**DOI:** 10.1038/s41598-017-05114-5

**Published:** 2017-07-14

**Authors:** Alessandro Chiarucci, Simone Fattorini, Bruno Foggi, Sara Landi, Lorenzo Lazzaro, János Podani, Daniel Simberloff

**Affiliations:** 1 Department of Biological, Geological and Environmental Sciences, University of Bologna, Via Irnerio 42, 40126 Bologna, Italy; 2 Department of Life, Health and Environmental Sciences, University of L’Aquila, via Vetoio, Coppito, 67100 L’Aquila, Italy; 3 CE3C – Centre for Ecology, Evolution and Environmental Changes/Azorean Biodiversity Group and Universidade dos Açores - Departamento de Ciências e Engenharia do Ambiente, Angra do Heroísmo, Açores, Portugal; 4 Department of Biology, University of Florence, Via G. La Pira, 4, 50121 Florence, Italy; 5 Department of Plant Systematics, Ecology and Theoretical Biology, Institute of Biology, L. Eötvös University, Pázmány P. s. 1/c, H-1117 Budapest, Hungary; 6 MTA-ELTE-MTM Ecology Research Group, Budapest, Hungary; 7 Department of Ecology and Evolutionary Biology, University of Tennessee, Knoxville, TN 37996 USA; 8 Department of Science for Nature and Environmental Resources, University of Sassari, Via Piandanna 4, 07100 Sassari, Italy

**Keywords:** Biodiversity, Biogeography

## Abstract

Although islands are model systems for investigating assembly of biological communities, long-term changes in archipelago communities are not well understood because of the lack of reliable data. By using a vast amount of floristic data we assembled a dataset of the plant species occurring on 16 islands of the Tuscan Archipelago, Italy, across two periods, 1830–1950 and 1951–2015. We collected 10,892 occurrence records for 1,831 species. We found major changes in the island plant assemblages between the two periods, with native flora significantly decreasing (−10.7%) and alien flora doubling (+132.1%) in richness. The species–area relationships demonstrated the scale-dependence of the observed changes for native and alien species. The observed floristic changes were dependent on island area, with smaller islands displaying high variability in richness and compositional changes and larger islands having more stable species assemblages. The richness of species associated with open landscapes, that had been maintained for centuries by traditional practices, markedly reduced while the number of woody species, associated with afforestation processes and invasion by alien woody plants, significantly incresed. These results demonstrate the great power of floristic studies, often available in grey literature, for understanding long-term biotic changes in insular ecosystems.

## Introduction

Islands can be viewed as sites of natural experiments in which biotic assemblages and ecological processes are clearly delimited by geographical constraints. Moreover, community structure is generally simpler on islands than on the mainland, making it easier to test specific ecological or evolutionary hypotheses. For these reasons, islands have long served as an inspiration and test bed for hypotheses on long-term evolutionary processes and large-scale assembly rules^1^.

Island biology received considerable impetus in the 1960s from the Equilibrium Theory of Island Biogeography (ETIB^2, 3^), which suggested theoretical bases for the development of evolutionary and ecological models^4^. According to the ETIB, the number of species that inhabit an island is a result of a balance between extinction and colonization rates. Because of these processes, the number of species inhabiting an island that does not physically change should remain relatively stable through time, but species composition can vary. Rapid arthropod recolonization of mangrove and cordgrass islands after artificial defaunation provided experimental support for this model^5, 6^. More recently, patterns of temporal turnover on relatively long temporal scales have come into focus, with studies depicting changes in species composition of island floras for periods varying between 10 and 40 years^7–9^. However, testing community processes in island ecosystems on longer temporal scales is difficult because of the lack of reliable biological data. Even in the paradigmatic case of the Krakatau islands, great caution was suggested in interpreting colonization and extinction rates because the use of a limited data set led to overestimated extinction rates^10^. Thus, it is not surprising that there has been virtually no research on long-term changes in island biotas, with a few remarkable exceptions such as the studies performed on Staten Island^11^ and the Stockholm archipelago^12^. On Staten Island, plant censuses conducted between 1879 and 1991 demonstrated important changes in species composition, with a significant reduction in the original (native) flora and a major increase in the number of non-native species^11^. In the Stockholm archipelago, land-use change was recognized as a major driver of temporal shift in plant species composition^12^. In fact, anthropogenic activities can magnify ecological and biological changes in island biotas in several ways, with various processes acting simultaneously on both species richness and composition^13^. Thus, islands that have experienced a long presence of human settlements are expected to have heavily transformed biotas, with parts of their past biogeographical legacy erased by anthropogenic pressures. In particular, persistent anthropogenic impacts are expected to reduce the number of native species, to facilitate the spread of non-native species, to strengthen the species-area relationship, and to weaken the species-isolation relationship^13^.

The Mediterranean basin was the cradle of some of the world’s most ancient civilizations, with many islands colonized from the first age of navigation. They thus offer opportunities for investigating the impact of long-term anthropogenic pressures on biotic communities^14^. The islands of the Tuscan archipelago, located in the Tyrrhenian Sea between peninsular Italy and Corsica, have a long history of human colonization. Thanks to its proximity to the universities of Pisa and Florence, this archipelago has also been subject to intensive and continuous botanical explorations in the last two centuries. For example, the papers by Sommier^15, 16^ on the Tuscan Archipelago were considered examples of the earliest floristic studies devoted to a group of small islands^17^. This exceptionally fortunate state of affairs led to the accumulation of an enormous amount of botanical data that can be used to trace changes undergone by the archipelagic flora. In this paper, we use this rich and unique amount of information to assess how insular biotas changed their floristic composition as a consequence of major land use changes on the islands after the Second World War. We hypothesized that the major transformations in habitat structure and land use on the islands of the Tuscan Archipelago after the Second World War have resulted in major changes of the archipelagic flora. In particular, we tested the following hypotheses based on the ETIB:

1. Under ETIB assumptions, island species composition changes over time, but species number should remain substantially similar. Thus, we tested if species richness in the Tuscan Archipelago was similar between the two study periods (1830 to 1950 and 1951 to 2015, see methods for explanations) characterized by strong changes in anthropic pressures. If human-induced changes in plant community structure were similar to those that can be expected under the ETIB assumption of natural turnover, local extinction due to natural and anthropic factors should be “balanced” by an increase in native and alien species, so overall species richness should remain similar in the two study periods.
2. Although competition as a key mechanism generating species turnover was only implicit in the original “core” ETIB^4^, MacArthur and Wilson emphasized the importance of competition^3^, and much subsequent research in ecology and biogeography has focused on competition between similar species as a key cause of island extinctions^1^. If species turnover indeed leads species to be replaced by others that are ecologically similar, we expect no substantial changes in the proportion of plant functional types between periods, even with large changes in species composition.
3. In an equilibrium scenario, we expect that the island species-area relationship (ISAR; i.e., the increase in species richness with increasing island area) should not change substantially between the two periods. By contrast, if human-induced changes differed from those associated with natural turnover, we expect that the ISAR of native and alien plants should differ substantially between the two study periods. In particular, if turnover is mainly due to anthropization, we expect a reduction in the slope of the native ISAR and an increase in the slope of the alien ISAR.
4. If species composition changed mainly by natural turnover, we expect that beta diversity (i.e. inter-island dissimilarity) and nestedness (i.e., the ordered variation in both species richness and species incidence, with the floras of smaller islands being subsamples of those of larger ones) should not vary substantially between the two periods. By contrast, if turnover was strongly influenced by extinction due to anthropization, we expect increased beta diversity (because of the loss of species from single islands) and decreased nestedness (because of a decrease in the ordered variation in species distribution across islands).
5. We expect that, under ETIB assumptions, natural turnover in species composition should be higher on islands that are too small to host stable populations over long periods (a phenomenon known as the Small Island Effect, SIE^1^).

## Results

In total, we collected 10,892 occurrence data for 1,831 sub-generic *taxa* (hereafter referred to as “species”, for simplicity) from 16 islands in two study periods: 5,714 for the first period (1830 to 1950) and 5,178 for the second period (1951 to 2015). The total number of species recorded in the first period was 1,601 and in the second period was 1,541. The two periods shared 1,311 species, yielding a Jaccard similarity index of 0.72. Thus, 28% of the species recorded in the total data set were found in only one of the two periods; 290 species disappeared and 230 species appeared in the archipelagic flora, from the first to the second period.

A slight reduction in the number of recorded species between the two periods (−3.7%) was due to a substantial decline in the number of native species (which decreased from 1,523 to 1,360, −10.7%), only partially balanced by an increase in the number of alien species (from 78 to 181, +132.1%), which conflicts with the ETIB (see Hypothesis 1, that is, overall species richness should remain similar through time). The proportion of alien species doubled from the first period (when they accounted for 4.9% of the total species richness) to the second one (when they represented 11.5% of the total species richness), indicating a major invasion by alien plants. The proportions of native and alien species differed substantially between the two periods (G-test, G = 50.191, df = 1, p < 0.0001), and the proportions of functional types (annual herbaceous, perennial herbaceous and woody species) also changed significantly (G = 6.600, df = 2, p = 0.037), but only because of an increase in the woody species (from 17.2% to 20.8%); changes in annual herbaceous species (from 39.7% to 38.2%) and perennial herbaceous species (from 43.1% to 41.1%) were negligible. This is consistent with Hypothesis 2, that the proportion of plant functional types should remain similar between periods, even with large changes in species composition.

**Figure 1:**
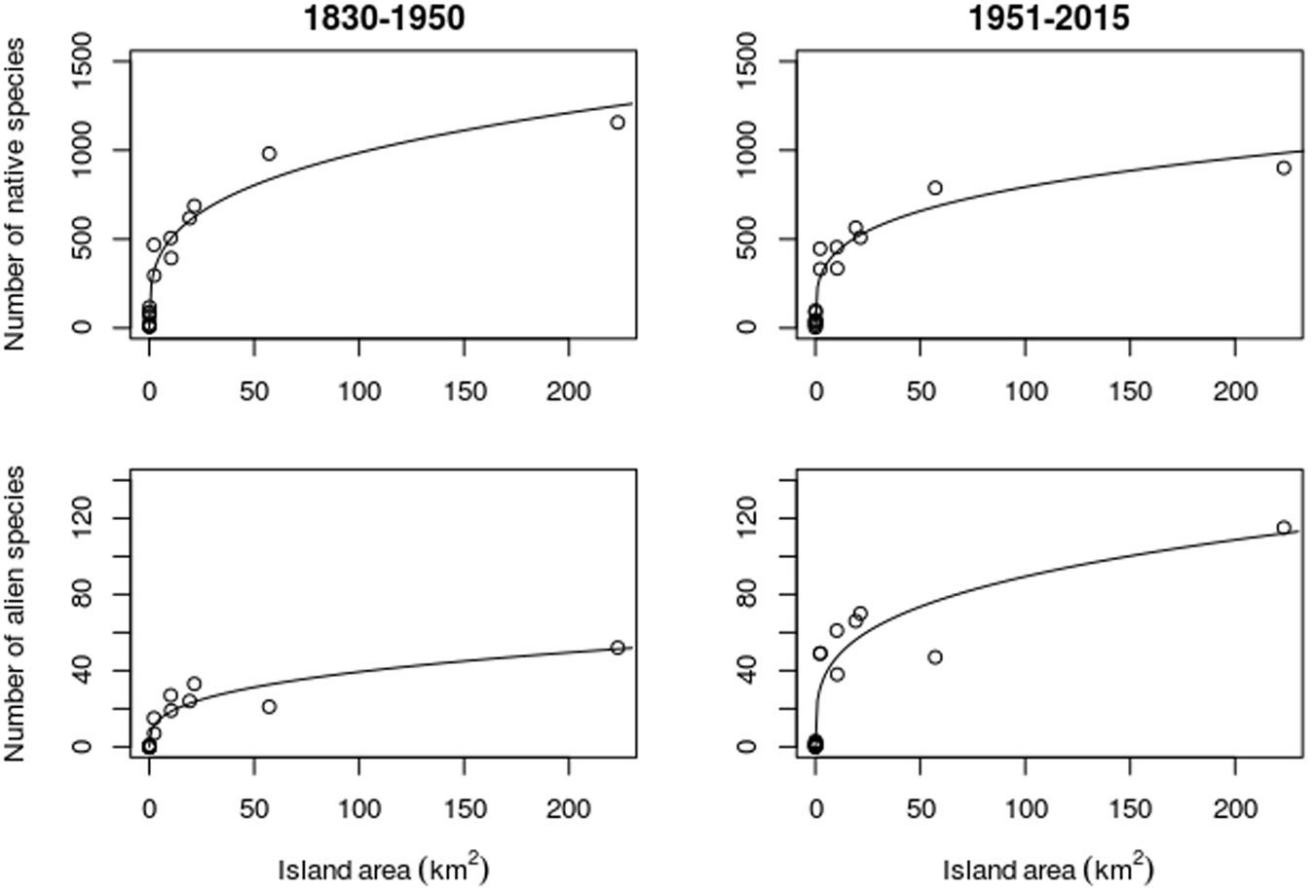
Island Species-Area Relationship, based on the Arrhenius model (*S* = *C·A* ^*z*^), calculated for plant species richness of the islands of the Tuscan Archipelago in the first period (1830–1950, left panels) and in the second period (1951–2015, right panels) for native (upper panels) and alien (lower panels) species, respectively.

The ISAR models for the native and alien species in the two periods showed different patterns, with the curve of native species flattening and the curve of alien species rising between the two periods (Fig. 1). This shift is produced by the combination of *c* and *z* values for the two curves. In particular, the *c* parameter (which expresses the expected number of species on a theoretical island with an area of 1 km^2^) showed opposite temporal changes between native and alien species, dropping from 252.2 to 229.3 for the former and increasing from 8.5 to 24.4 for the latter (Table 1), which contrasts with ETIB expectations of no substantial changes (see Hypothesis 3). This indicates a decrease in the number of native species and a much higher increase in the richness of alien species for an island of unit area between the two periods. On the other hand, the *z* parameter (which is a scaling factor describing how strongly species richness responds to area changes along the ISAR curve) declined for both native and alien species, indicating that the rate of species accumulation with increasing island area declined for both groups of species (Table 1). In sum, the ISAR of native species decreased globally, while the ISAR of alien species rose, especially at smaller spatial scales. It is striking that the fossil island Monte Argentario (which is currently connected to the mainland by two low strips of sand) shows a large positive residual in the two ISAR curves of the native species, but a notable negative residual in the alien species ISARs. These residuals indicate a higher species richness of native species and a lower richness of alien species with respect to the ISAR expectations for this peculiar island with “zero isolation”.

**Table 1:** Curve-fitting for the Island Species-Area Relationship (ISAR), based on the Arrhenius’ power function, calculated for plant species richness values of the Tuscan Archipelago islands (n = 16).

Group of species	Year	Model fitting and parameters			
		c	z	Achieved convergence tolerance	F
*Native plants*	1830–1950	252.2***	0.296***	2.00E-006	94364.8
	1951–2015	229.3***	0.269***	7.97E-006	83223.3
*Alien plants*	1830–1950	8.5***	0.332***	4.17E-006	397.1
	1951–2015	24.4***	0.282***	9.03E-006	2660.4
*Annual herbaceous plants*	1830–1950	136.9***	0.257***	3.48E-006	27054.8
	1951–2015	122.3***	0.228***	8.15E-006	35458.3
*Perennial herbaceous plants*	1830–1950	78.8***	0.359***	8.72E-006	17591.0
	1951–2015	77.1***	0.328***	1.28E-006	14904.9
*Woody plants*	1830–1950	43.2***	0.296***	1.61E-006	4010.6
	1951–2015	51.2***	0.282***	2.13E-006	2462.6

Parameters (c and z) of the ISAR models are shown as well as the achieved convergence tolerance and F-value. Separate models were calculated for different groups of species based on their origin (native and alien) and functional types (annual herbaceous, perennial herbaceous, and woody) and for each study period. Significance codes: ***p < 0.001; **p < 0.01; *p < 0.05.

**Figure 2:**
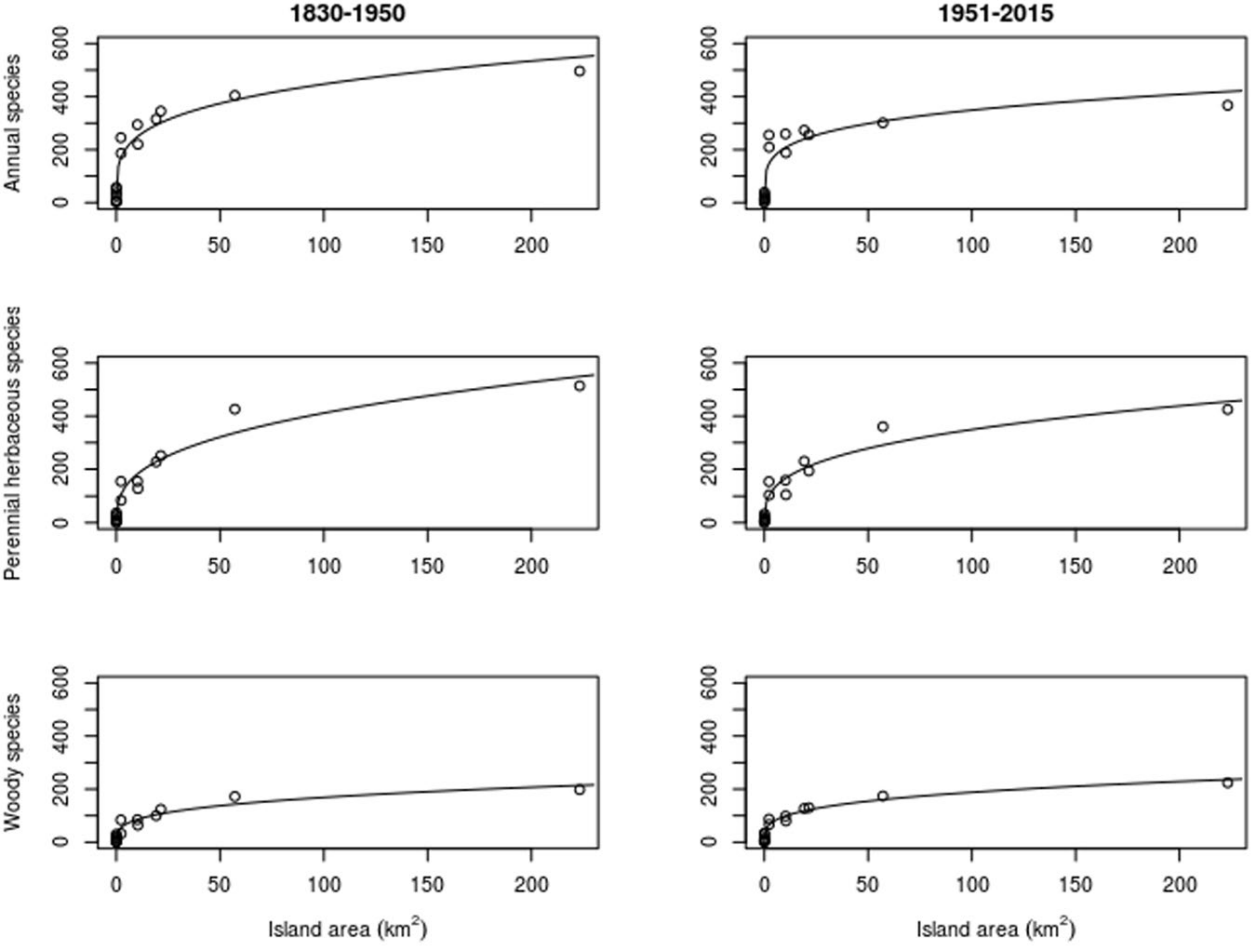
Island Species-Area Relationship, based on the Arrhenius model (*S* = *C·A* ^*z*^), calculated for plant species richness of the islands of the Tuscan Archipelago for the first period (1830–1950, left panels) and for the second period (1951–2015, right panels) for annual herbaceous (upper panels), perennial herbaceous (middle panels), and woody (lower panels) species, respectively.

The ISAR models constructed for the two periods followed different patterns in the three functional groups (Fig. 2). Curves of annual and perennial herbaceous species showed a general flattening, whereas the woody species curves showed a slightly increased steepness. The *c* parameter showed a marked decrease for the annual herbaceous plants, a slight reduction for the perennial herbaceous plants, and an evident increase for the woody plants (Table 1). The *z* values decreased in all functional groups.

**Figure 3:**
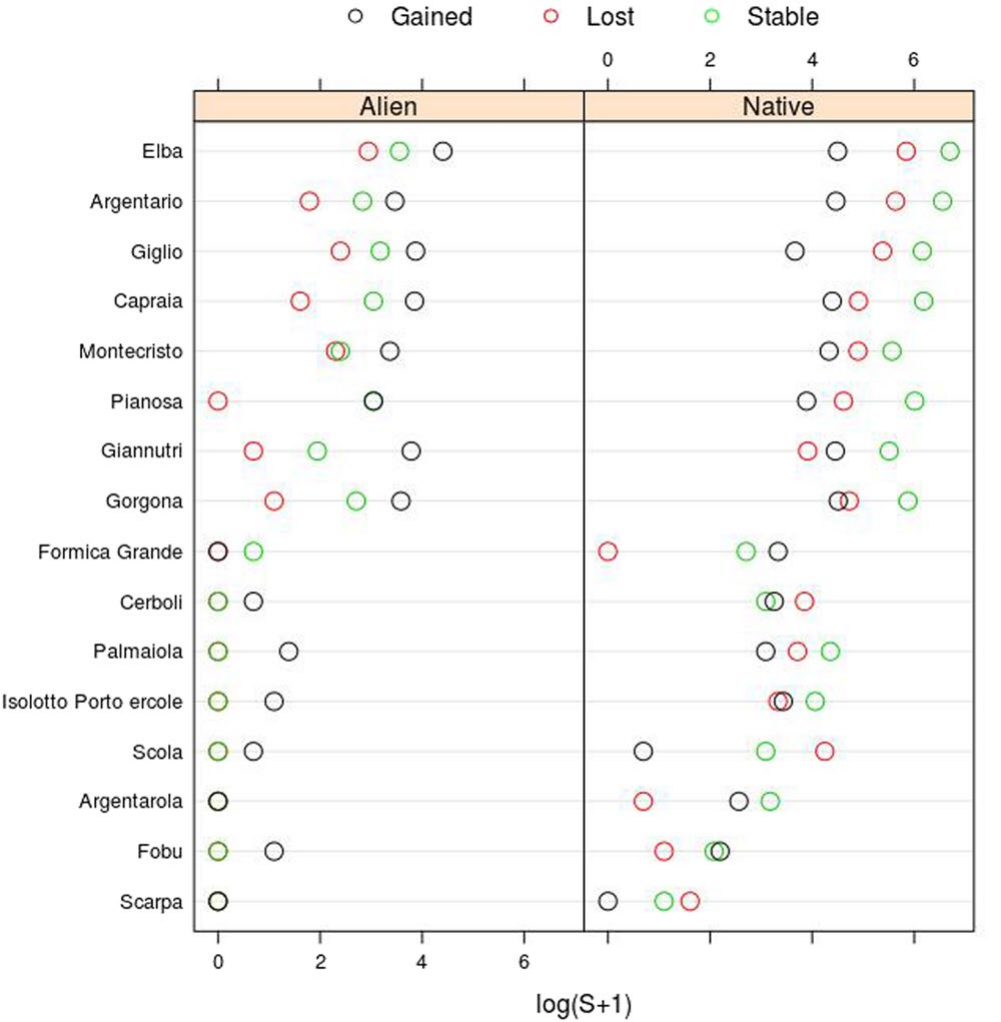
Number of alien and native species that remained stable (green), were gained (black), or were lost (red) in the flora of each island from the first period (1830–1950) to the second period (1951–2015).

**Figure 4:**
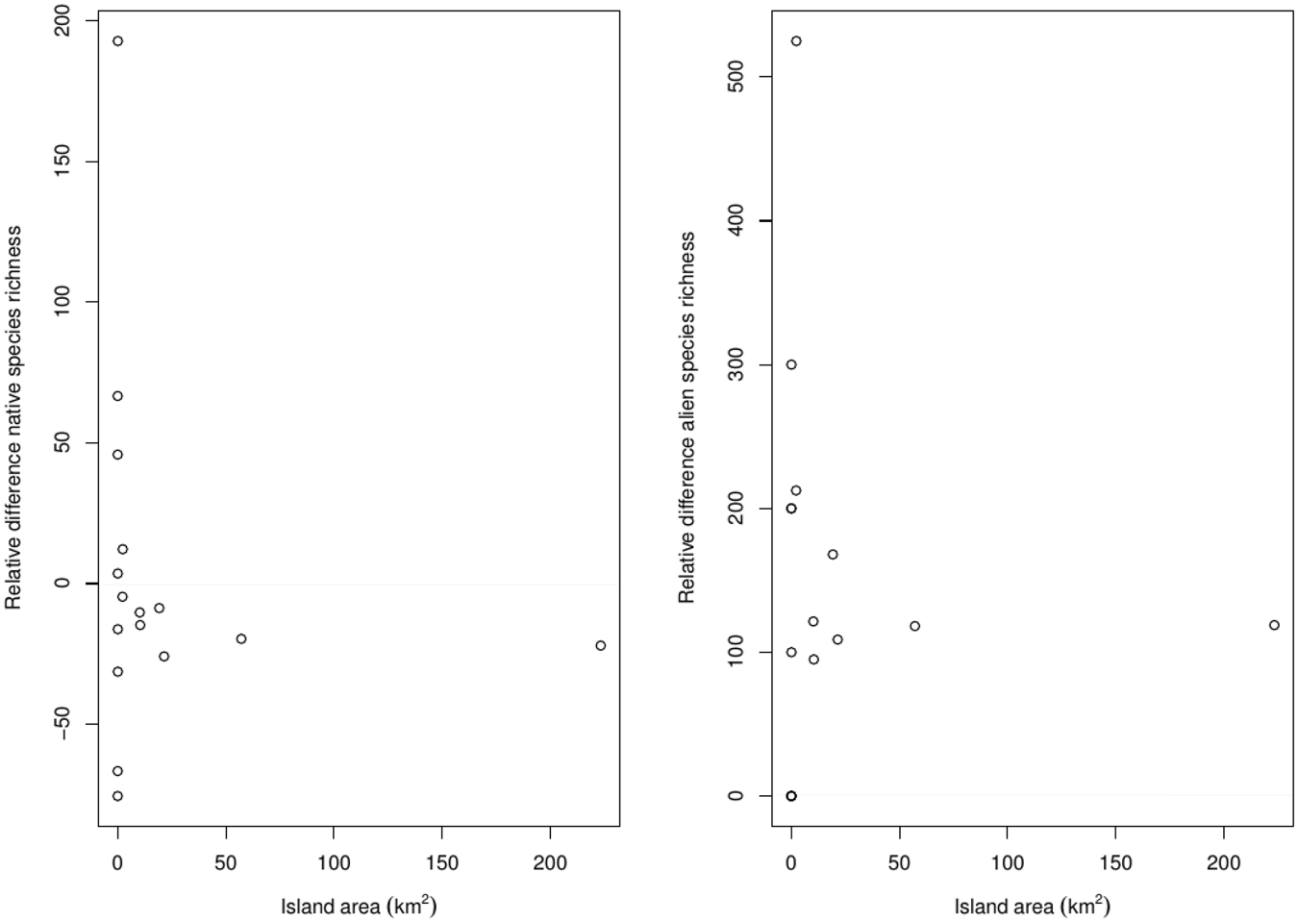
Relative changes in species richness of the first period (1830–1950) with respect to the second period (1951–2015) on each island of the Tuscan archipelago in relation to island area (n = 16) for native (left panel) and alien (right panel) species. Note that for the alien species graph some data points have identical values and thus overlap.

Mean plant species richness per island in the second period was 6.0% lower than in the first one (Fig. 3). Linear regressions of island species richness between the two periods showed an intercept value higher than 0 for both alien and native species (Table 2), suggesting an increase in species richness of these two groups for the smallest islands. The slope of 0.797 for the linear regression for the native species indicates a global reduction in their species richness per island, while the slope of 2.173 for the alien species indicates their richness increase per island. Relative changes in native species richness per island showed a general decrease (with most values negative), but with high variation for the smallest islands (Fig. 4), some of which had, in fact, a substantial increase in the number of native species. A similar funnel–like pattern was also found for alien species but with globally positive values. In this case, none of the islands had a negative relative value (i.e., a reduction in alien species richness). The proportion of alien species in the flora of each island increased from an average value of 2.3% in the first period to 6.6% in the second one.

**Table 2:** Results of the ordinary least squares regression (OLS) for the number of species recorded on each island in the 1951–2015 period regressed on the number of species recorded on the same islands in the 1830–1950 period (n = 16).

	OLS statistics			OLS parameters	
	R^2^	AdjR^2^	P-value	Intercept	Slope
Native species	0.983	0.9821	<0.0001	14.30	0.798
Alien species	0.934	0.9288	<0.0001	4.00	1.790

**Figure 5:**
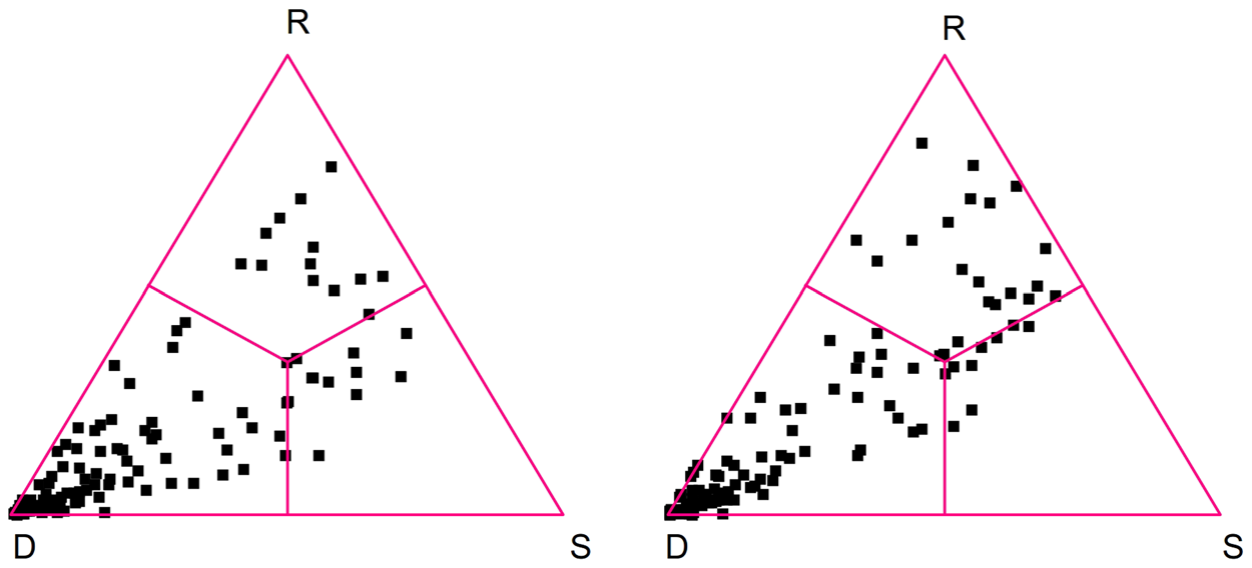
Simplex plots based on Similarity (S), Richness Difference (D) and Species Replacement (R) for all the pairwise comparisons (n = 120) of the 16 islands of the Tuscan archipelago in the 1830-1950 (left) and 1951–2015 (right) periods, respectively.

Similarly to the reduction in species richness, the filling of the species-by-island presence/absence matrix showed a loss of species occurrences between the two periods, falling from 22% to 20% (Table 3), in agreement with the global reduction of island occurrences. The SDR Simplex analyses for the two study periods gave similar results, because the beta diversity patterns were dominated by the strong differences in species richness between islands and islets (Table 3, Fig. 5). As a consequence of a decrease in similarity and an increase in species replacement, beta diversity showed a slight increase between periods and matrix nestedness a clear decrease, which conflicts with ETIB predictions that beta diversity and nestedness should be stable through time (see Hypothesis 4). This is confirmed by the nestedness matrix analysis, which resulted in a NODF value of 65.537 and an RN value of 0.866 (Z = 55.770, p < 0.001) for the first period, and a NODF value of 57.121 and an RN value of 0.751 (Z = 47.063, p < 0.001) for the second one, thus indicating a decrease in nestedness.

**Table 3:** Results of the Simplex SDR analyses for all pairwise (n = 120) comparisons of the 16 islands of the Tuscan archipelago in the two study periods.

	1830–1950	1951–2015
Matrix fill	22.31%	21.00%
*S – Similarity*	15.09%	14.16%
*D – Richness Difference*	68.98%	66.86%
*R – Species Replacement*	15.93%	18.98%

**Figure 6:**
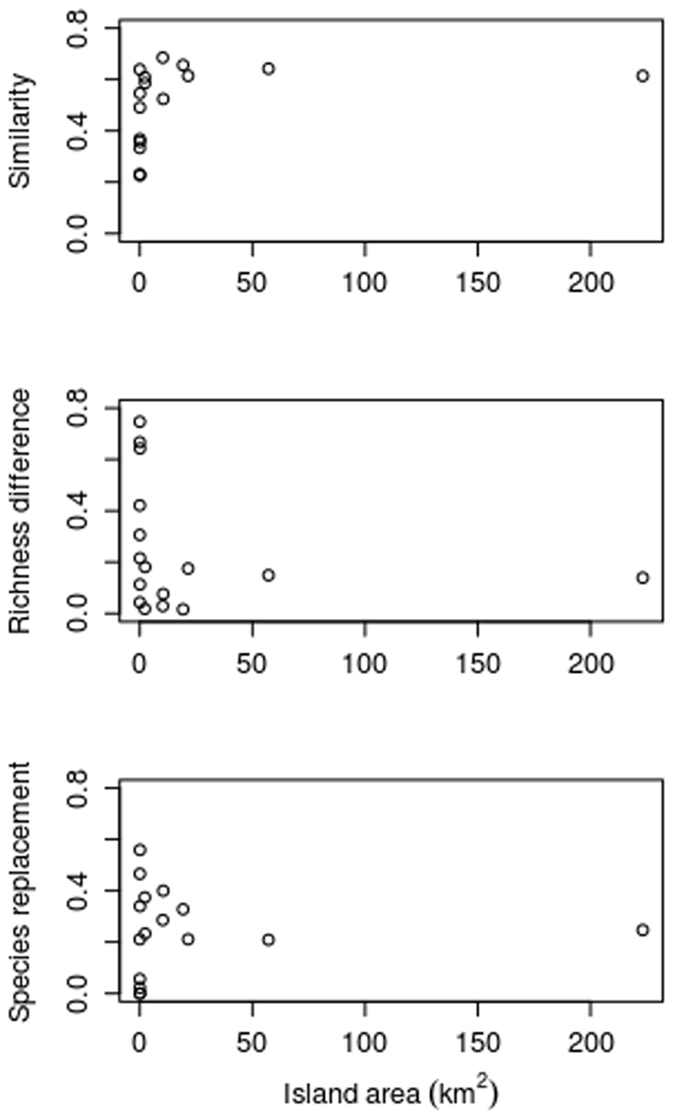
Changes of each island flora in the second period (1951–2015) with respect to the first period (1830–1950) for cross-temporal similarity (S), relative richness difference (D), and relative species replacement (R) in relation to island area.

Among islands, changes in species similarity, replacement, and richness were influenced by island area (Fig. 6). In particular, cross-temporal similarity (*S*, i.e., how similar each island was to itself across the two time periods) varied greatly among smaller islands but reached a sort of threshold value of about 0.6 for islands larger than 10–20 km^2^. Relative richness difference (*D*) showed similar high variation for small islands and a relatively low value (about 0.2) for islands larger than 10–20 km^2^. Finally, relative species replacement (*R*) showed a funnel-like pattern, with a high dispersion of values for smaller islands and a constant value of about 0.2 for islands larger than 10–20 km^2^. This picture globally indicates that the larger the island, the higher the proportion of the flora that did not change, that is consistent with the Small Island Effect of the ETIB (see Hypothesis 5).

## Discussion

Despite the simple theoretical assumptions of the ETIB^1–4^, surprisingly few studies have experimentally tested it. Also, these studies have often used data for few islands or short time periods^7–9^, whereas tests at large spatial and temporal scales are still lacking, likely because of difficulties in obtaining high quality data for entire archipelagos and long time periods. In the present paper, we were able to assemble data from a set of islands spanning over five orders of magnitude in size and that were sampled for two centuries, and demonstrate how the flora of the Tuscan archipelago islands changed dramatically in species richness and composition in this period.

In general, data assembled from multiple sources for large scale and long term analyses have variable degrees of completeness, which makes their analysis difficult^18^. For the flora of the Tuscan archipelago, however, the plant occurrence data were collected by experienced botanists affiliated with the major universities of the region and their quality can be considered very high in terms of geographic coverage, spatial resolution, and taxonomic identification. We must also consider the fact that the first investigated period exceeds the second one, and this could have led to the accumulation of more species. However, the two study periods were both characterized by similarly intense investigation efforts which allowed us to base comparisons on very large datasets for both periods. Moreover, the data on land use change available for the island of Elba showed that major changes, such as the reduction of agricultural areas and increase of forest area and settlements, happened only in the last 5–6 decades, and land use was relatively stable for a long previous period^19, 20^.

The flora of the Tuscan archipelago islands dramatically changed in species richness and composition in the last two centuries, with only 72% of the plant species found in both study periods, and the numbers of colonising species and extinct species indicate very high turnover rates. However, in contrast to the ETIB predictions^4^ (see Hypothesis 1), the total number of species did not remain stable but showed a substantial reduction, with a loss of about 5% of total species and 11% of native species. This species loss in the original flora was only partly balanced by the enormous increase (about 95%) in alien species.

A general trend towards an increase of alien flora is indeed common to most small Mediterranean Islands^21^. The current percentage of alien species in the Tuscan Archipelago flora is close to values recorded from similar insular contexts in the Mediterranean basin. It is similar to the value reported for Sicily (12.6%)^22^, but appears lower than those of other Mediterranean archipelagos, such as the Balearic Islands (19%)^23^ and other major islands such as Sardinia and Corsica (18% and 17% of their total flora, respectively^24^). The proportion of alien species in the total flora reaches very high values even on very remote islands. For example, in 1997 alien plants constituted 45% of the total flora of the Galápagos Islands and their richness on the various islands increased between +28% and +61% in the period from 1987 to 1995^25^.

A dramatic increase in the proportion of alien species has also been documented for Staten Island (New York) for the period 1879–1991^11^. However, that study considered only a single island, whereas we analysed changes for the various islands that form an archipelago and for the archipelago as a whole. This allowed us to investigate how island area may affect species loss and compositional changes. This in an important point, because natural turnover in species composition is expected to be particularly high on islands that are too small to host stable populations over long periods (Small Island Effect, SIE; see Hypothesis 5). For example, important changes in plant species composition have been documented from 1908 to 2008 in the Stockholm archipelago (Sweden)^12^, the islands of which ranged between 0.3 and 33 *ha* and were hence probably unable to maintain stable populations for most species.

The concept of SIE has been subject to strong criticism^26, 27^, but recent papers^28, 29^ have defended the idea of greater biotic instability on small islands, as originally proposed by MacArthur & Wilson^1, 3^. In the Tuscan Archipelago, relative changes in native species richness per island showed high variability on the smallest islands, which supports the idea that ecological and biogeographical processes acting on small islands are less predictable than those on larger islands^28^. Similarly, we found that turnover in species composition varied greatly for islands smaller than 10–20 km^2^, providing further support for the notion that the compositional dynamics on smaller islands are especially difficult to predict.

We observed important floristic changes not only for the whole archipelago but also at the level of single islands, as demonstrated by the shift in the parameters of the species–area relationship and by differences in species richness, which contrasts with ETIB predictions^1, 3, 4^ (see Hypothesis 3). On average, each island lost more than 20% of its native flora but gained many alien species, which almost doubled in number, as indicated by the slope of the regression models. Aggemyr & Cousins^12^ observed an overall increase (31%) in the cumulative flora of the Stockholm archipelago but no significant increases in species richness per unit area, which can be explained by a parallel increase in the mean island area determined by isostatic uplift that occurred during the same temporal interval. Similarly to the human-induced changes reported for Staten Island^11^ and for the Stockholm archipelago^12^, the islands of the Tuscan archipelago experienced major land-use changes. After the Second World War, and more intensively after the 1950s, abandonment of most traditional agricultural practices and the spread of tourism as a major economic activity^19, 20, 30^ greatly transformed the terrestrial ecosystems of the Tuscan islands. The abandonment of agricultural practices led to a strong reduction in availability of agricultural areas, which for some islands showed a reduction of 72% of the surface occupied until 1950^19, 20^. Other open areas, such as pastures, also decreased and were largely transformed into forests^20^ that are less suitable for annual and perennial herbaceous plants.

In addition to changes in species richness, the Tuscan islands flora underwent significant changes in species composition and assemblage structure (see Hypotheses 4 and 5), although the proportion of functional types changed only slightly (Hypothesis 2). The proportion of alien species on each island doubled between the two periods. In general, island biotas are extremely prone to invasion by alien plant species following anthropogenic transformations^31–34^. Because of long-term human exploitation, natural habitats of Mediterranean islands are affected by prolonged, intense, and varied stress factors that favor invasion of alien species^34^. The Tuscan archipelago illustrates this phenomenon, with some alien species already reported as highly invasive and needing urgent management^32^.

The increase in the proportion of alien species, albeit particularly impressive, was not the only change that affected the floristic composition of the Tuscan islands as a result of long-term changes in land-use. In particular, two processes clearly emerged in the data, namely reduction in the number of herbaceous annual species and increase in the number of woody species. The major reduction in the number of herbaceous annual species per island is certainly due to the almost complete abandonment of agricultural and pastoral practices and the consequent reduction of agricultural areas, which have been re-colonized by woody species and then converted into forest formations^19, 20^. The increase in the number of woody species is also connected to the increase in the number of alien species, since most of the alien species that invaded the Tuscan islands are woody^32^. Natural afforestation is a major process that occurred on these islands, as documented for the island of Giannutri following land abandonment^35^.

In conclusion, the use of traditional data, such as the information stored in specialized regional literature (a precious but typically overlooked source of information) to reconstruct long-term changes in biotic communities^36–38^ allowed us to draw, for the first time, a high-quality picture of changes that occurred in plant assemblages of an entire archipelago over a very long period in response to prolonged anthropization. Our findings partially disagree with expectations that can be derived from the ETIB^2, 3^ and that have played a pivotal role in diverse areas such as the design of natural reserves and the prediction of extinction rates^1^. However, we emphasize that the island habitats and land use underwent major transformations in the investigated period^19, 20, 30^, thus violating the ETIB basic assumption of no physical change. As a consequence, we detected major changes not only in plant species composition but also in species richness for both single islands and the whole archipelago. The major changes in land use and management practices of the islands are likely to have obscured the changes that might have been predicted by the ETIB and point to the need to consider land use change as a primary driver for the biotic changes in the island and archipelagic floras. Also, our findings indicate that land-use transformations and invasion processes by alien species are phenomena that make biogeographical dynamics of this island system more complex than expected on the basis of the ETIB. These results are particularly relevant because major biotic transformations, including, for example, negative effects on coastal ecosystems, increased frequency of fires, and major vegetational changes are also expected on Mediterranean islands because of climate change^39^. In particular, the negative effects of climate change on the native forest vegetation^39^, in conjunction with the spreading of alien woody species, would exacerbate the future transformation of island vegetation.

## Methods

The Tuscan archipelago includes seven larger and mainly continental (land bridge) islands, several smaller islets, and the large palaeo-island of Monte Argentario, which was isolated before the formation of two low strips of sand, Tombolo della Feniglia (with sand accumulated by sea currents), and Tombolo della Giannella (with sediments transported by the nearby Albegna river), which connect it with the adjacent mainland (see supplementary Table S1 for details). After 1950, many Mediterranean islands experienced major land use changes due to a shift from the traditional economy based on pastoralism, forestry, and agriculture to a new economy based on tourism^19, 40–42^. For example, land use changes occurring on Elba, the largest in the study islands, included a strong reduction of agricultural activities, an increase of forest coverage, and the expansion of residential areas^19, 20^. About 32 km^2^ of agricultural areas were lost, corresponding to about 72% of the agricultural areas that were used for centuries up to the period immediately following the Second World War^20^.

All available published papers and some unpublished sources (such as masters’ theses and doctoral dissertations and technical reports) dealing with the plants of the Tuscan archipelago were searched and compiled by the botanical team of the University of Florence (which was the most important botanical institution in Italy for a long period, hosting the largest Italian herbarium, the Italian Botanical Society, and a library where most of the botanical literature about Italy is archived). These references were checked to extract occurrence records for all those species reported as spontaneous on at least one of the studied islands. Each occurrence datum represented the record of a single species on a single island in one of the two periods, without taking into account the number of reports. The complete list of data sources used in this paper is reported in supplementary Table S2. Plant species that were clearly reported only as cultivated were not considered in this study. All occurrence data present in the sources were checked in light of present knowledge, and the nomenclature was standardized to a current taxonomy in order to make data collected over such a long period directly comparable. Overall, we assembled the existing data on plant species occurrences on 16 islands (7 major islands, Monte Argentario fossil island, and 8 islets) in two main periods: from 1830 to 1950 and from 1951 to 2015. The complete data set assembled for the present study is reported in supplementary Table S3. We used 1950–1951 as a pivotal shift date because of the major changes in the human presence and activities on the islands from the 1950s, when most of the archipelago’s economy shifted from traditional agriculture to tourism.

To glean information about the invasion process, we classified species as native (*N*) or alien (*A*) on the basis of national and regional data^43–46^. To detect changes in functional types, we initially assigned each species to one of Raunkiaer’s life forms^47^. Then, to reflect the relationship between functional groups and major habitat type, we grouped these life forms into the following broader categories: annual herbaceous species (AH, typical of dry Mediterranean vegetation or heavily disturbed habitats), perennial herbaceous species (PH, associated with stable open landscapes and largely pastures), and woody species (W, typical of maquis and forest habitats).

We modelled the Island Species-Area Relationship (ISAR^48^) using the Arrhenius power function^49^ (1):

1 $$S=c\cdot {A}^{z}$$

where *S* is the number of species recorded on each island, *A* is the island area, *c* and *z* are fitted parameters that express the number of species per unit area (*c*) and the increment of the number of species with increase in island area (*z*), respectively^50^.

Although several mathematical functions have been proposed to model the ISAR, comparative studies identify the power function as the model that most frequently fits empirical data best (at least for island systems^48, 51^) and that is best supported by ecological theories^52, 53^, but see Harte *et al*.^54^). Model fitting was performed by using the non–linear modelling procedure of the *SSArrhenius* function contained in the “*vegan*” *R* package^55^. We modelled ISARs for the total flora as well as for native (*N*) and alien (*A*) species and the three functional groups (*AH*, *PH*, *W)* separately. We conducted separate analyses for each of the two study periods.

In order to test for general patterns of temporal changes, the species richness values of *N* and *A* species recorded on each island in the second period were compared with the values recorded in the first period, by linear regression, under the null expectation of 0 and 1 values for the intercept and the slope, respectively (i.e., no change in species richness on each island, as predicted by the ETIB). Then, the change in *N* and *A* species richness for each island from the first to the second period, measured by the relative residuals of the linear regression model, was related to island area.

Between-island beta diversity was quantified, partitioned, and visualized for each study period by means of the SDR (Similarity–richness Difference–Replacement) simplex approach^56^ for presence/absence matrices. This involves calculating three relativized indices for each pair of islands, as detailed below.

Similarity (*S*) was calculated using Jaccard’s coefficient^57^ (2):

2 $$S=\frac{{S}_{ij}}{{S}_{t}}$$

where: *S*_*ij*_ is the number of species shared by islands *i* and *j* and *S*_*t*_ is the pooled species richness of the same pair of islands.

Relative richness difference (*D*) was calculated as the ratio of the absolute difference between the species richness of island *i* (*S*_*i*_) and that of island *j* (*S*_*j*_) to the total number of species, *S*_*t*_, (3):

3 $$D=\frac{|{S}_{i}-{S}_{j}|}{{S}_{t}}$$

Finally, relative species replacement (*R*) was calculated by equation (4):

4 $$R=2\cdot \frac{min\{{S}_{i}-{S}_{ij},{S}_{j}-{S}_{ij}\}}{{S}_{t}}$$

*D* and *R* are additive components of pairwise dissimilarity or beta diversity, while *S* is complementary to them, so that these three quantities always sum to 1.0^56^. This allows the use of a simplex or ternary plot, in which each vertex corresponds to one index (*S*, *D*, or *R*), and each point represents a pair of islands in a position determined by the values of these three indexes.

In addition, the above three complementary coefficients were also used to evaluate changes in the floristic composition of each island between the two periods. In this case, *S*_*t*_ was the cumulative species richness for a given island in the two periods, *S*_*ij*_ the number of species occurring in both periods on that island, and relative richness difference and relative species replacement were interpreted similarly, for the two periods on the same island. The relationship between the three components and island area was then depicted graphically. Calculations were performed with the computer program SDR Simplex^56^.

Finally, we explored how nestedness of the whole set of islands varied between the two periods, by using the NODF index (nestedness measure based on overlap and decreasing fill) for the binary species-by-site matrix. This nestedness statistic was calculated separately for islands (NODF columns, which tests whether depauperate assemblages constitute subsets of progressively richer ones) and for species (NODF rows, which tests whether less widespread species are found in subsets of the sites where the most widespread species occur), then combined for the whole matrix (NODF matrix). To assess significance of nestedness, we calculated Z-values based on 100 null matrices constructed using the CE (proportional row totals, proportional column totals) randomization algorithm, which assigns to each cell an occupancy probability proportional to the corresponding row and column totals^58^. This means that the probability that an island will be colonized is proportional to the species richness of the island (which in turn reflects its size, habitat diversity, carrying capacity, proximity to the mainland, etc.) and species frequency (which may be considered to indicate its colonization capability).

Z-values were calculated as [*Nr-*mean(*Ns*)]/stdev(*Ns*), where *Nr* is the nestedness of the matrix under study, and mean(*Ns*) and stdev(*Ns*) are, respectively, the average and standard deviation of the nestedness values of the null matrices^59, 60^. Although Z-values can be used to assess whether a matrix is significantly nested or not, they cannot provide information about the ‘magnitude’ of nestedness^61^. For this purpose, we also calculated ‘relative nestedness’ (RN)^62^, which is computed as [*Nr-*mean(*Ns*)]/mean(*Ns*). For each matrix, values of NODF rows (and associated Z values and RN) were virtually identical to those of the NODF matrix and very similar to those of NODF columns. Thus, for simplicity, we report only values of the NODF matrix, which express the “overall” matrix nestedness.

Nestedness analyses were conducted using the NeD program^63^.

## Electronic supplementary material

Supplementary Table S1

Supplementary Table S2

Supplementary dataset
