# Author Correction: Plant recording across two centuries reveals dramatic changes in species diversity of a Mediterranean archipelago

**DOI:** 10.1038/s41598-019-54272-1

**Published:** 2019-12-05

**Authors:** Alessandro Chiarucci, Simone Fattorini, Bruno Foggi, Sara Landi, Lorenzo Lazzaro, János Podani, Daniel Simberloff

**Affiliations:** 1 Department of Biological, Geological and Environmental Sciences, University of Bologna, Via Irnerio 42, 40126 Bologna, Italy; 2 Department of Life, Health and Environmental Sciences, University of L’Aquila, via Vetoio, Coppito, 67100 L’Aquila, Italy; 3 CE3C – Centre for Ecology, Evolution and Environmental Changes/Azorean Biodiversity Group and Universidade dos Açores - Departamento de Ciências e Engenharia do Ambiente, Angra do Heroísmo, Açores Portugal; 4 Department of Biology, University of Florence, Via G. La Pira, 4, 50121 Florence, Italy; 5 Department of Plant Systematics, Ecology and Theoretical Biology, Institute of Biology, L. Eötvös University, Pázmány P. s. 1/c, H-1117 Budapest, Hungary; 6 MTA-ELTE-MTM Ecology Research Group, Budapest, Hungary; 7 Department of Ecology and Evolutionary Biology, University of Tennessee, Knoxville, TN 37996 USA; 8 Present Address: Department of Science for Nature and Environmental Resources, University of Sassari, Via Piandanna 4, 07100 Sassari, Italy

Correction to: *Scientific Reports* 10.1038/s41598-017-05114-5, published online 14 July 2017

The Supplementary Table S1 file that accompanies this Article contains a typographical error in Table S1. The area value for ‘Elba’ incorrectly reads as ‘2244.09’, this should read as ‘223.241’.

