# Supplementary material for: Plant recording across two centuries reveals dramatic changes in species diversity of a Mediterranean archipelago
Source: Sci Rep. 2017 Jul 14;7:5415. doi: 10.1038/s41598-017-05114-5 (PMC5511228; doi:10.1038/s41598-017-05114-5)
Supplement: Supplementary file 1 — Supplementary Table S1 [file 41598_2017_5114_MOESM1_ESM.docx]

**Table S1**. Physical data of the 16 islands of the Tuscan archipelago included in this study: latitude and longitude (WGS84, decimal degrees), island area, elevation of the highest point of the island (Altitude), main geological substrates within the island (Substrates), nearest larger island within the archipelago (Nearest island).

| **Island** | **Latitude** | **Longitude** | **Area (*km^2^*)** | **Elevation (*m*)** | **Substrates** | **Nearest island** | **Distance to nearest island (*km*)** | **Distance to mainland (*km*)** | **Prevalent habitat types** | **Notes** |
| --- | --- | --- | --- | --- | --- | --- | --- | --- | --- | --- |
| Gorgona | 43.430932 | 9.899021 | 2.26 | 255 | Metamorphic | Capraia | 39 | 35 | pine woods, shrublands, rocky shores |  |
| Capraia | 43.038575 | 9.81988 | 19.32 | 447 | Trachytes | Elba | 34 | 52 | shrublands, rocky slopes, rocky shores, temporary ponds | *31 km from Corsica |
| Palmaiola | 42.865538 | 10.47457 | 0.083 | 83 | Sandstone | Elba | 3.14 | 7.3 | shrublands, rocky shores |  |
| Cerboli | 42.858345 | 10.547543 | 0.089 | 83 | Limestone | Elba | 8.7 | 6.9 | shrublands, rocky shores |  |
| Elba | 42.778187 | 10.192739 | 2244.09 | 1018 | Granites, Metamorphic, Limestone, Sandstone | Pianosa | 13.81 | 9.89 | holm oaks woods, pine woods, shrublands, grasslands, rocky slopes, rocky shores |  |
| Scarpa | 42.622071 | 10.079953 | 0.005 | 15 | Limestone, Sandstone | Pianosa | 0.27 | 57 | shrublands, rocky shores |  |
| Scola | 42.583811 | 10.106143 | 0.015 | 34 | Limestone, Sandstone | Pianosa | 0.26 | 57.2 | shrublands, rocky shores |  |
| Pianosa | 42.581037 | 10.077738 | 10.28 | 27 | Limestone, Sandstone | Elba | 13.81 | 57.66 | pine woods, shrublands, rocky shores, grasslands, |  |
| Formica Grande | 42.576747 | 10.881488 | 0.099 | 11 | Limestone | Giglio | 21.12 | 14.98 | shrublands, rocky shores |  |
| Argentarola | 42.418538 | 11.081376 | 0.012 | 20 | Limestone | Monte Argentario | 0.57 | 13.81 | shrublands, rocky shores |  |
| Monte Argentario | 42.407271 | 11.150397 | 60.23 | 635 | Limestone, Shales | Giannutri | 11.6 | 4.76* | holm oak woods, shrublands, rocky shores | *connected to mainland by 2 sand strips |
| Isolotto Porto Ercole | 42.381389 | 11.211111 | 0.063 | 67.5 | Granites, Metamorphic, Limestone | Monte Argentario | 0.33 | 6.5 | shrublands, rocky shores |  |
| Formica di Burano | 42.366667 | 11.316667 | 0.0072 | 10 | Limestone | Monte Argentario | 8.86 | 4.25 | shrublands, rocky shores |  |
| Giglio | 42.353631 | 10.901604 | 21.54 | 498 | Granites, Limestone | Monte Argentario | 14.9 | 26.5 | shrublands, rocky shores |  |
| Montecristo | 42.337762 | 10.308529 | 10.43 | 645 | Granites | Pianosa | 30.69 | 61.2 | shrublands, rocky slopes, rocky shores, temporary ponds |  |
| Giannutri | 42.252801 | 11.100881 | 2.39 | 93 | Limestone, Sandstone | Monte Argentario | 11.5 | 22.5 | shrublands, rocky shores |  |
