# Supplementary material for: Plant recording across two centuries reveals dramatic changes in species diversity of a Mediterranean archipelago
Source: Sci Rep. 2017 Jul 14;7:5415. doi: 10.1038/s41598-017-05114-5 (PMC5511228; doi:10.1038/s41598-017-05114-5)
Supplement: Supplementary file 2 — Supplementary Table S2 [file 41598_2017_5114_MOESM2_ESM.docx]

**Table S2**. List of sources used to assemble the check-lists of species for each island of the Tuscan archipelago in the two considered period (1830-1950 and 1951-2015).

**Papers used to compile the check-list of plant species in the Tuscan archipelago islands for the period 1830-1950**

Arcangeli, G. Le piante fino ad ora raccolte in Gorgona. *Ric. Lav. Ist. Bot. R. Univ. Pisa* **2**: 109–145. (1888).

Baroni, E. *Supplemento generale al Prodromo della Flora Toscana di Teodoro Caruel*. 638 pagg (Societa’ Botanica Italiana 1897-1908).

Béguinot, A. Notizie botaniche su alcune erborazioni invernali attraverso le isole dell’arcipelago toscano. *Bull. Soc. Bot. Ital.* **1901**, 44–56 (1901).

Bolzon, P. Appunti sulla flora dell’Elba. *Riv. Ital. Nat.* **11**, 63-66 (1891).

Bolzon, P. Contributo alla flora dell’Elba. *Riv. Ital. Nat.* **12**, 45-47, 85-86 (1892).

Bolzon, P. Contributo alla flora della Pianosa. *Bull. Soc. Bot. Ital.* **1892**, 257–261 (1892).

Bolzon, P. Contributo alla flora dell’Elba. *Bull. Soc. Bot. Ital.* **1892**, 311-314, 356-361 (1892).

Bolzon, P. Erborizzazione all’Isola dell’Elba. Centurie Prima-Quinta + Appendice. *Bull. Soc. Bot. Ital.* **1893**, 23–31; 166–173; 237–243; 306–313; 350–357; 411–418 (1893).

Bolzon, P. Seconda contribuzione alla Flora di Pianosa. *Bull. Soc. Bot. Ital.* **1893**,164–166 (1893).

Caruel, T. *Prodromo della Flora Toscana*. (Ed. Le Monnier 1860).

Caruel,T. Statistica botanica della Toscana. *Giorn. Bot. Ital.* **1**, 243-283 (1864).

Caruel, T. Florula di Montecristo. *Atti Soc. Ital. Sci. Nat*. **6**, 74-109 (1864).

Levier, E. & Sommier S. Addenda ad Floram Etruriae. *N. Giorn. Bot. Ital*. **23**, 241-270 (1991).

Moris, J. & De Notaris J. Florula Caprariae. *Mem. R. Accad. Sci. Torino, ser. 2*, 2: 1-244.

Negri, G. 1950 Escursione della Societa’ Botanica Italiana all’ Isola d’Elba (Aprile 1950): appunti sulla vegetazione. *N. Giorn. Bot. Ital. n.s.* **57**, 276-293 (1839).

Requien, G. Catalogo delle piante che non trovo nella flora di Caprariae che ho riportato da quest’isola. *Giorn. Bot. Ital.* 2, 116 (1852).

Savi, G. Due centurie di piante appartenenti alla flora etrusca. Pisa (1804).

Savi, P. Florula Gorgonica. *Giorn. Bot. Ital.* **1**, 243-283 (1844).

Sommier, S. Una erborazione all’isola del Giglio, in marzo. *Bull. Soc. Bot. Ital*. **1894**, 128–134 (1894).

Sommier, S. Seconda erborazione all’isola del Giglio, in maggio. *Bull. Soc. Bot. Ital*. **1894**, 245–249 (1894).

Sommier, S. La microflora mediterranea precoce ed alcuni appunti sulla flora di Giannutri. *Bull. Soc. Bot. Ital*., **1897**, 122-129 (1897).

Sommier, S. Piante vascolari nuove raccolte a Giannutri dal 3 al 7 marzo 1897. *Bull. Soc. Bot. Ital.* **1897**, 129–136 (1897).

Sommier, S. Aggiunte alla florula di Capraia. *N. Giorn. Bot. Ital*., n.s., **5**, 106-139 (1898).

Sommier, S. Resoconto di una gita botanica nell’Arcipelago Toscano. *Boll. Soc. Bot. Ital*., **1898**, 126-137 (1898).

Sommier, S. Piante raccolte durante la gita sociale alla Gorgona. *Bull. Soc. Bot. Ital*. **1899**, 117–126 (1899).

Sommier, S. Aggiunte alla flora dell'Elba. *Bull. Soc. Bot. Ital*., **1900**, 204-212 (1900).

Sommier, S. *L'Isola del Giglio e la sua Flora* (Ed. Clausen 1900).

Sommier, S. Nuove aggiunte alla flora dell'Elba. *Bull. Soc. Bot. Ital*., **1900**, 340-344 (1900).

Sommier, S. Cenni sulla flora di Pianosa. *Bull. Soc. Bot. Ital.* **1901**, 298–307.

Sommier, S. Di una specie di Statice dell’Arcipelago Toscano. *N. Giorn. Bot. Ital*. n.s. **9**, 210-213 (1902).

Sommier, S. La Carex grioletti Roem. Nell’Isola del Giglio. *Bull. Soc. Bot. Ital.* **1902**, 203-207 (1902).

Sommier, S. La flora dell’Arcipelago Toscano. *N. Giorn. Bot. Ital*., n.s., **9**, 319-354 (1902).

Sommier, S. La flora dell’Arcipelago Toscano. Nota II. *N. Giorn. Bot. Ital*. n.s. **10**, 133-200 (1903).

Sommier, S. Nuove aggiunte alla flora di Giannutri. *Bull. Soc. Bot. Ital*. **1900**, 228-232 (1903).

Sommier, S. Aggiunte alla flora del Monte Argentario e nuove stazioni di Carex grioletti. *Bull. Soc. Bot. Ita.* 1903, 232-236 (1903).

Sommier, S. Alcune piante recentemente raccolte all’Elba, non ancora indicate per quest’isola. *Boll. Bot. Ital*. **1904**, 304-305 (1904).

Sommier, S. La flora dell’Isola di Pianosa nel Mar Tirreno. *N. Giorn. Bot. Ital*. n.s **16**, 357-438 (1909).

Sommier, S. La flora dell’Isola di Pianosa nel Mar Tirreno. *N. Giorn. Bot. Ital*. n.s **17**, 123-164 (1910).

Sommier, S. Nuovi materiali per la Flora del Monte Argentario. *Bull. Soc. Bot. Ita*. **1911**, 37-41 (1911).

Sommier, S. Ulteriore contributo alla Flora del Monte Argentario. *N. Giorn. Bot. Ita*. n.s. **19**, 116-123 (1912).

Tanfani, E. Florula di Giannutri. *N. Giorn. Bot. Ital*. n.s. 22, 153-216 (1890).

Thie’baut De Berneaud, A. *Voyage a l’ Isle d’Elba, suivi d’une notice sur les autres isles de la Mer Tyrrhenienne*. (Colas 1808, Trad. Akademos & LIM. 1993).

**Papers used to compine the check-list of plant species in the Tuscan archipelago islands for the period 1951-2015**

Arrigoni, P.V. & Viegi, L. *La flora vascolare esotica spontaneizzata della Toscana*. 216 pagine (Regione Toscana 2011).

Baldini, R.M. Florula delle isole Formiche di Grosseto (Arcipelago Toscano). *Webbia* **44**, 271-278 (1990).

Baldini, R.M. Flora delle Isole satelliti del Monte Argentario (Arcipelago Toscano). *Webbia* **46**, 107- 123(1991).

Baldini, R.M. Flora vascolare dell'Isola del Giglio (Arcipelago Toscano): revisione tassonomica ed aggiornamento. *Webbia* **52**, 307-404 (1998).

Baldini, R.M. Flora vascolare dell’Isola di Pianosa (Arcipelago Toscano): revisione tassonomica ed aggiornamento. *Webbia* **55**, 107-189 (2000).

Baldini, R.M. Flora vascolare dell’Isola di Giannutri (Arcipelago Toscano). *Webbia* **56**, 69-125 (2001).

Baldini, R.M. & Luccioli, E. Segnalazioni Floristiche Italiane. *Inf. Bot. Ital.* 22, **69** (1990).

Baldini, R.M. & Tosi, G. Segnalazioni Floristiche Italiane. *Inf. Bot. Ital.* 22, **68** (1990).

Bussotti, L., Garbari, F. & Nannoni, R. Le stazioni di Chamaerops humilis L. (Arecaceae) nell’Arcipelago Toscano. *Atti Soc. Tosc. Sci. Nat. Mem. Ser. B* **103**, 115-118 (1997).

Carlesi, V. & Peruzzi, L. Notulae 1650-1664 in Notulae alla checklist della flora vascolare italiana – (eds Nepi, C., Peccenini, S. & Peruzzi, L. **9**) *Inform. Bot. Ital.* **42**, 377-379 (2010).

Carta, A. Notula: Notula 1712 in Notulae alla checklist della flora vascolare italiana – (eds Nepi C., Peccenini S. & Peruzzi L. **10**) *Inform. Bot. Ital.* **42**, 519-520 (2010).

Carta, A. Contributi 1-14 in Contributi per una flora vascolare di Toscana - (eds Peruzzi, L., Viciani, D. & Bedini, G. **1-85**) *Atti Soc. Tosc. Sci. Nat. Mem. Ser. B* **116**, 34-35 (2010).

Carta, A. Notulae 95-102 in Contributi per una flora vascolare di Toscana. I - (eds Peruzzi, L., Viciani, D. & Bedini, G. **86-142**). *Atti Soc. Tosc. Sci. Nat. Mem. Ser. B* **117**, 24-25 (2011).

Carta, A., Ferretti, G., Foggi, B., Guidi, T., Lazzaro, L., Siccardi, E. & Viciani D. Contributi 172-177 in Contributi per una flora vascolare di Toscana - (eds Peruzzi, L., Viciani, D. & Bedini, G. **143-180**) *Atti Soc. Tosc. Sci. Nat. Mem. Ser. B* 118, **42** (2012).

Carta, A., Forbicioni L., Frangini G. & Peruzzi L. Notula 1369 in Notulae alla checklist della flora vascolare italiana (eds Nepi, C., Peccenini, S. & Peruzzi, L. **4**) *Inform. Bot. Ital.* **39**: 421-422 (2007).

Carta, A. & Frangini, G. Contributi 24-26 in Contributi per una flora vascolare di Toscana - (eds Peruzzi, L., Viciani, D. & Bedini, G. **1-85**) *Atti Soc. Tosc. Sci. Nat. Mem. Ser. B* **116**, 36 (2010).

Carta, A., Frangini, G., Gestri, G., Lazzeri, V., Peruzzi, L. & Pierini, B. Contributo 158 in Contributi per una flora vascolare di Toscana - (eds Peruzzi, L., Viciani, D. & Bedini, G. **143-180**) *Atti Soc. Tosc. Sci. Nat. Mem. Ser. B* **118**, 40 (2012).

Carta, A., Gestri, G., Mannocci, M., Pierini, B. & Peruzzi L. Contributo 27 in Contributi per una flora vascolare di Toscana - (eds Peruzzi, L., Viciani, D. & Bedini, G. **1-85**) *Atti Soc. Tosc. Sci. Nat. Mem. Ser. B* **116**, 36 (2010).

Carta, A., Gestri, G. & Peruzzi L. Contributi 15-18 in Contributi per una flora vascolare di Toscana - (eds Peruzzi, L., Viciani, D. & Bedini, G. **1-85**) *Atti Soc. Tosc. Sci. Nat. Mem. Ser. B* **116**, 35 (2010).

Carta, A., Gestri, G., Peruzzi, L. & Pierini B. Contributi 28-30 in Contributi per una flora vascolare di Toscana - (eds Peruzzi, L., Viciani, D. & Bedini, G. **1-85**) *Atti Soc. Tosc. Sci. Nat. Mem. Ser. B* **116**, 36 (2010).

Carta, A., Guidi, T., Lazzaro, L., Siccardi, E., Viganò, R. & Ferretti G. Contributi 217-223 in Contributi per una flora vascolare di Toscana - (eds Peruzzi, L., Viciani, D. & Bedini, G. **181-246**) *Atti Soc. Tosc. Sci. Nat. Mem. Ser. B* **119**, 27 (2013).

Carta, A. & Peruzzi L. Notulae 1488-1489 in Notulae alla checklist della flora vascolare italiana - (eds Nepi, C., Peccenini, S. & Peruzzi L. **6**) *Inform. Bot. Ital.* **40**, 253-254 (2008).

Carta, A., Pierini, B. & Peruzzi, L. Contributo 86 in Contributi per una flora vascolare di Toscana - (eds Peruzzi, L., Viciani, D. & Bedini, G. **86-142**) *Atti Soc. Tosc. Sci. Nat. Mem. Ser. B* **117**, 24 (2011).

Carta, A., Pierini, B. & Peruzzi, L. Contributo 178 in Contributi per una flora vascolare di Toscana - (eds Peruzzi, L., Viciani, D. & Bedini, G. **143-180**) *Atti Soc. Tosc. Sci. Nat. Mem. Ser. B* **118**, 42 (2012).

Cioffi, V., Ferretti, G. & Foggi B. Contributi 73-75. In: Peruzzi L., Viciani D., Bedini G. (eds) Contributi per una flora vascolare di Toscana. I (1-85). *Atti Soc. Tosc. Sci. Nat. Mem. Ser. B* **116**, 40 (2010).

Del Prete, C. & Tosi, G. Orchidee spontanee dell'Argentario ( ed. A.T.L.A. 1981).

Del Prete, C. & Tosi, G. Contributi alla conoscenza delle Orchidaceae d' Italia. X. *Atti Soc. Tosc. Sci. Nat. Mem. Ser. B* **88**, 217-224 (1982).

Del Prete, C., Tichy, H. & Tosi, G. *Le orchidee spontanee della Maremma grossetana*. (PRO.GRA. ed. Massa 1993).

Fabbri, F. Per una flora dell’isola di Montecristo. Le piante vascolari raccolte da A. Chiarugi nel maggio del 1957. *Giorn. Bot. Ital.* **70**, 629-633 (1963).

Ferretti, G. & Foggi B. Contributi 245-246 in Contributi per una flora vascolare di Toscana - (eds Peruzzi, L., Viciani, D. & Bedini, G. **181-246**) *Atti Soc. Tosc. Sci. Nat. Mem. Ser. B* **119**, 29 (2013).

Ferretti, G., Lazzaro, L., Foggi, B. & Romolini R. Contributi 316-317 in Contributi per una flora vascolare di Toscana - (eds Peruzzi, L., Viciani, D. & Bedini, G. **247-319**) *Atti Soc. Tosc. Sci. Nat. Mem. Ser. B* 120, **42** (2014).

Ferretti, G., Lazzaro, L., Giuliani, C. & Foggi B. Secondo contributo alla conoscenza della flora esotica dell'Arcipelago Toscano, Italia. *Atti Soc. It. Sci. Nat. Museo Civ. Stor. Nat. Milano* **154**, 115-130 (2013).

Ferretti, G., Mannocci, M., Mazzoncini, V., Fiorini, G., Foggi, B. & Viciani D. *Indagini sistematiche su Saxifraga granulata e S. corsica in Arcipelago Toscano. Primi risultati.* Atti Convegno Gruppo Floristica Società Botanica Italiana. Roma, 18-19 ottobre 2013: 45-47 (2013).

Ferretti, G., Mannocci, M., Mazzoncini, V., Fiorini, G., Foggi, B. & Viciani D. *Systematic investigations concerning Saxifraga granulata s.l. in the Tuscan Archipelago (Northern Tyrrhenian Sea): evidences for describing new taxa in Capraia and Montecristo islands*. Poster 109° Congresso della Società Botanica Italiana. Firenze, 2-5 settembre 2014 (2014).

Filipello, S. & Sartori, F. La vegetazione dell'Isola di Montecristo (Arcipelago Toscano). *Atti Ist. Bot. Lab. Crittog. Univ. Pavia Ser.* **6**, 113-202 (1983).

Foggi, B., Cartei, L., Pignotti, L., Signorini, M.A., Viciani, D., Dell’Olmo, L. & Menicagli, E. 2006 Il paesaggio vegetale dell’Isola d’Elba (Arcipelago toscano): studio fitosociologico e cartografico. *Fitosociologia*, **43** (1) – Suppl. 1: 3-94.

Foggi, B., Cartei, L. & Pignotti L. La vegetazione dell'Isola di Pianosa (Arcipelago Toscano, Livorno). *Braun-Blanquetia* **43**, 3-41 (2008).

Foggi, B. & Grigioni, A. Contributo alla conoscenza della vegetazione dell’Isola di Capraia (Arcipelago toscano). *Parlatorea* **3**, 5-33 (1999).

Foggi, B., Grigioni, A. & Luzzi P. La flora vascolare dell’Isola di Capraia (Arcipelago toscano): aggiornamento, aspetti fitogeografici e di conservazione. *Parlatorea* **5**, 5-53 (2001).

Foggi, B., Guidi, T., Capecchi, M., Baldini, R.M. & Grigioni, A. Biological Flora of the Tuscan Archipelago islets (Tyrrenian Sea). *Webbia* **64**, 23-45 (2009).

Foggi, B., Signorini, M.A., Grigioni, A. & Clauser, M. 2000 La vegetazione di alcuni isolotti dell’Arcipelago toscano. *Fitosociologia* **37**, 69-91.

Foggi, B. & Venturi, E. Contributo 79 in Contributi per una flora vascolare di Toscana - (eds Peruzzi, L., Viciani, D. & Bedini, G. **1-85**) *Atti Soc. Tosc. Sci. Nat. Mem. Ser. B* **117**, 41 (2010).

Forbicioni, L., Frangini, G., Pierini, B., Ferretti, G., Foggi, B., Giuliani, C. & Lazzaro, L. Contributo 261 in Contributi per una flora vascolare di Toscana - (eds Peruzzi, L., Viciani, D. & Bedini, G. **247-319**) *Atti Soc. Tosc. Sci. Nat. Mem. Ser. B* **120**, 37 (2014).

Fossi Innamorati, T. La flora vascolare dell'Isola d'Elba (Arcipelago Toscano). Prima parte. *Webbia* **36**, 273-411 (1983).

Fossi Innamorati, T. La flora vascolare dell'Isola d'Elba (Arcipelago Toscano). Parte seconda. *Webbia* **43**, 201-267 (1989).

Fossi Innamorati, T. La flora vascolare dell'Isola d'Elba (Arcipelago Toscano). Parte terza. *Webbia* **45**, 137-185 (1991).

Fossi Innamorati, T. La flora vascolare dell'Isola d'Elba (Arcipelago Toscano). Parte quarta. *Webbia* **49**, 93-123 (1994).

Fossi Innamorati, T. Addenda ed emendanda alla flora vascolare dell'Isola d'Elba (Arcipelago Toscano). *Webbia* **51**, 385-389 (1997).

Frangini, G. & Carta, A. Notula 103 in Contributi per una flora vascolare di Toscana - (eds Peruzzi, L., Viciani, D. & Bedini, G. **86-142**) *Atti Soc. Tosc. Sci. Nat. Mem. Ser. B* **117**, 25 (2011).

Frangini, G. & Carta, A. Notula 171 in Contributi per una flora vascolare di Toscana - (eds Peruzzi, L., Viciani, D. & Bedini, G. **143-180**) *Atti Soc. Tosc. Sci. Nat. Mem. Ser. B*, **118**, 42 (2012).

Galasso, G., Gestri, G., Peruzzi, L. & Banfi E. Notula: 69 in Notulae alla checklist della flora vascolare italiana - (eds Nepi, C., Peccenini, S. & Peruzzi L. **4**) *Inform. Bot. Ital.* **43**, 147 (2011).

Gargano, M.L., Mandracchia, G. & Venturella, G. Contributo alla conoscenza del genere Tamarix L. nell'Isola del Giglio (Arcipelago Toscano). *Inform. Bot. Ital.* **41**, 125-128 (2009).

Gonnelli, V., Carta, A., Zoccola, A., Landi, M., Viciani, D., Ferretti, G., Quilghini, G., Parri, F., Giombini, R. & Marsiaj G. Contributi 179-180 in Contributi per una flora vascolare di Toscana - (eds Peruzzi, L., Viciani, D. & Bedini, G. **143-180**) *Atti Soc. Tosc. Sci. Nat. Mem. Ser. B* **118** 43-44 (2012).

Gori, C. *Inventario floristico ed analisi fitogeografica delle isole dell'Arcipelago Toscano*. Univ. Studi Firenze. Tesi di Dottorato (1993).

Gramuglio, G. Resoconto di una erborizzazione nell’Isola di Giannutri (Arcipelago Toscano). *Giorn.Bot. Ital*. **73**, 175- 181 (1966).

Guiggi, A. Catalogo delle Cactaceae naturalizzate in Italia con osservazioni tassonomiche, nomenclaturali e corologiche. *Riv. Piem. St. Nat*. **29**, 103-140 (2008).

Guiggi, A. Aggiunte e correzioni al Catalogo delle Cactaceae naturalizzate in Italia. *Riv. Piem. St. Nat*. **31**, 35-54 (2010).

Iamonico, D. & Forbicioni, L. Contributo 91 in Contributi per una flora vascolare di Toscana - (eds Peruzzi, L., Viciani, D. & Bedini, G. **86-142**) *Atti Soc. Tosc. Sci. Nat. Mem. Ser. B* **117**, 24 (2011).

Iamonico, D., Forbicioni, L. & Frangini G. Notula 1505 in Notulae alla checklist della flora vascolare italiana (eds Nepi, C., Peccenini, S. & Peruzzi, L. **6**) *Inform. Bot. Ital*. 40, 258-259 (2008).

Lastrucci, L., Calamassi, R., Ferretti, G., Galasso, G. & Foggi B. Contributo alla conoscenza della flora esotica dell'Isola di Capraia (Arcipelago Toscano, Italia). *Atti Soc. It. Sci. Nat. Museo Civ. Stor. Nat. Milano* **153**, 127-134 (2012).

Lastrucci L., Foggi B., Mantarano N., Ferretti G., Calamassi R., Grigioni A., 2010 La vegetazione del laghetto "Lo Stagnone" (Isola di Capraia, Toscana). *Atti Soc. Tosc. Sci. Nat. Mem. Ser. B* **116** (2009): 17-25.

Lazzaro L., Ferretti G., Galasso G., Lastrucci L., Foggi B., 2013 Contributo alla conoscenza della flora esotica dell'Arcipelago Toscano, Italia. *Atti Soc. It. Sci. Nat. Museo Civ. Stor. Nat. Milano* **154**, 3-24.

Lazzaro, L., Ferretti, G. & Giuliani C. Contributi 351-355 in Contributi per una flora vascolare di Toscana - (eds Peruzzi, L., Viciani, D. & Bedini, G. **320-356**) *Atti Soc. Tosc. Sci. Nat. Mem. Ser. B*, **121**, 32-33 (2015).

Lazzaro, L., Ferretti, G., Giuliani, C. & Foggi B. A checklist of the alien flora of the Tuscan Archipelago (Italy) Webbia **69**, 157-176 (2014).

Lazzaro, L., Ferretti, G., Lastrucci, L. & Foggi B. Contributi 303-311 in Contributi per una flora vascolare di Toscana - (eds Peruzzi, L., Viciani, D. & Bedini, G. **247-319**) *Atti Soc. Tosc. Sci. Nat. Mem. Ser. B*. **120**, 41-42 (2014).

Lazzaro, L., Giuliani, C., Ferretti, G. & Guiggi A., Notula 200 in Notulae alla checklist della flora esotica italiana - (eds Barberis, G., Nepi, C., Peccenini, S. & Peruzzi L. **9**) *Inform. Bot. Ital.* **45**, 310 (2013).

Lazzaro, L., Gotti, C. & Ferretti, G. Contributo 356 in Contributi per una flora vascolare di Toscana - (eds Peruzzi, L., Viciani, D. & Bedini, G. **320-356**) *Atti Soc. Tosc. Sci. Nat. Mem. Ser. B* **121**, 33 (2015).

Lazzaro, L., Mariotti, D., Viciani, D., Benesperi, R. & Ferretti, G., Contributo 318 in Contributi per una flora vascolare di Toscana - (eds Peruzzi, L., Viciani, D. & Bedini, G. **247-319**) *Atti Soc. Tosc. Sci. Nat. Mem. Ser. B*, **120**, 42 (2014).

Lo Cascio, P. , Pasta, S. Floristic and ecological remarks on the Islet Formica di Burano (Tuscan Archipelago, Thyrrenian sea). *Atti Soc. Tosc. Sci. Nat. Mem. Ser. B* **116**, 45–48 (2010).

Lucchese, F. & Lattanzi, E. Segnalazioni Floristiche Italiane: 233. Iris lutescens Lam. *Inform. Bot. Ital.* **15**, 82 (1984).

Mannocci, M. Contributo alla conoscenza della flora dell’isola di Capraia (Livorno) 1. Galium minutulum Jordan, 1846. *Quad. Mus. St. Nat. Livorno* **1**, 28-32 (1980).

Mannocci, M. & Falconcini, E. Mentha requienii ssp. bistaminata. Nuova sottospecie dell’Isola di Capraia (Li- vorno). *Quad. Mus. St. Nat. Livorno* **6**, 37-41 (1986).

Mannocci, M. Segnalazioni Floristiche Italiane: 1105-1107. Salix atrocinerea Brot., Euphorbia segetalis All., Sedum brevifolium DC. *Inform. Bot. Ital.* **36**, 81 (2004).

Mannocci, M Notulae 1613-1616 in Notulae alla checklist della flora vascolare italiana - (eds Nepi, C., Peccenini, S. & Peruzzi, L. **8**) *Inform. Bot. Ital.* **41**, 356 (2009).

Mannocci, M. & Barsotti, G. Aggiunte alla flora dell’Isola di Capraia (Arcipelago toscano). *Quad. Mus. Stor. Nat. Livorno* **10**, 39-45 (1989).

Marsiaj, G., Avanzi, A., Benesperi, R., Castellani, B., Di Nuzzo, L., Lazzaro, L. & Ferretti G. Contributo 437 in Contributi per una flora vascolare di Toscana - (eds Peruzzi, L., Viciani, D. & Bedini, G. **357-439**) *Atti Soc. Tosc. Sci. Nat. Mem. Ser. B* **121**, 70 (2015).

Moggi, G., Rizzotto, M. & Gori, C. Aspetti significativi della flora dell’isola di Gorgona (Arcipelago Toscano), ai fini della sua protezione. *Atti Soc. Tosc. Sci. Nat. Mem. Ser. B*. **97**, 103–120 (1991).

Montelucci, G., Notule vegetazionali sulla Capraia. *Lav. Soc. Ital. Biogeogr*. **5**, 81-91 (1976).

Paoli, P. & Romagnoli, G. 1976 La flora vascolare dell'isola di Montecristo (Arcipelago Toscano). *Webbia* **30**, 303-456.

Peruzzi, L. & Carta, A. *Crocus ilvensis* sp. nov. (sect. *Crocus*, Iridaceae), endemic to Elba Island (Tuscan Archipelago, Italy). Nord. J. Bot. **29**, 6-13 (2011).

Ricceri, C. Holcus setiglumis Boiss. et Reut. specie nuova per l’Italia. *Webbia* **25**, 183-190 (1970).

Ricceri, C. & Rizzotto M. Entità nuove per la flora dell’ Isola di Giannutri (Provincia di Grosseto). *Boll. Soc. Sarda Sci. Nat.* 23, 161-164 (1984).

Rizzotto, M. Research on the genus Limonium (Plumbaginaceae) in the Tuscan Archipelago (Italy). *Webbia* **53**, 241-282 (1999).

Rizzotto, M. Flora of the Island of Gorgona (Tuscan Archipelago, Italy). *Webbia* **66**, 85-118 (2011).

Sabato, S. Note sulla flora e vegetazione di Pianosa (Arcipelago Toscano). *Webbia* **32**, 189-196 (1977).

Sartori F., Aggiunte alla Flora Vascolare dell'Isola di Montecristo (Arcipelago Toscano). *Atti Ist. Bot. Lab. Crittog. Univ. Pavia, Ser*. 6, **13**, 171-180 (1980).

Signorini, M.A. & Foggi, B. A survey of the genus *Festuca* L. (*Poaceae*) in Italy. VII. – *Festuca gamisansii* Kerguélen subsp. *aethaliae*, subsp. nov. Plant Biosys. **132**, 105-112 (1998).

Signorini, M. A. & Ricceri C. Trisetum corsicum Rouy (Gramineae), specie nuova per la flora italiana. *Webbia* **50**, 211-222 (1996).

Viegi, L. & Cela Renzoni, G. *Flora esotica d’Italia: le specie presenti in Toscana*. CNR Prog. Final. Promozione della qualità dell'ambiente (1981).
